# A life course perspective on determinants of discontinuance of active participation in sports activities

**DOI:** 10.1016/j.pmedr.2023.102402

**Published:** 2023-09-12

**Authors:** Xiaoyue Chen, Astrid Kemperman, Harry Timmermans

**Affiliations:** a Department of the Built Environment, Eindhoven University of Technology, Eindhoven, The Netherlands; b Department of Air Transportation Management, Nanjing University of Aeronautics and Astronautics, Nanjing, China

**Keywords:** Life course, Life transitions, Neighborhood characteristics, Sports discontinuance, Retrospective longitudinal data, A two-level binary logistic regression

## Abstract

Physical inactivity remains a global public health challenge today. Determining why people stop regularly participating in sports is significant to develop targeted intervention strategies for sports promotion and healthy living. As sports participation is dynamic throughout life, a life-course perspective is needed to provide a more comprehensive understanding. This study adopts a life-course perspective to explore the determinants of the change from active participation in sports to becoming inactive. Based on online retrospective survey data collected in the Netherlands, a two-level binary logistic regression model is estimated to capture the effects of socio-demographics, sports motivations, life transitions, and neighborhood characteristics on sports dropout over the lifespan. Results show that dropout from sports is age-specific, and that people are less likely to discontinue sports participation when they have health and weight loss goals. Life transitions have different effects. The cessation of living with physically active people appears to be the most important event to make people stop sporting, followed by having a baby, and then owning the first car. Compared with education-related events, work-related events are more likely to cause people to stop sporting. Moreover, the probability of sports discontinuance may increase when residents feel unsafe doing physical activities in their neighborhoods or when the neighborhood has sufficient greenspace for walking. The findings have implications for supporting sports participants to continue exercising by addressing the barriers.

## 1 Introduction

With great health benefits (Downward et al., 2016), participating in sports is a popular way of active living, particularly in the Netherlands. In 2020, 55% of the Dutch population aged over 4 participated in sports weekly, one of the highest levels of participation in the world (Duijvestijn et al., 2021). Despite the popularity of sports, high drop-out rates were observed, especially among adolescents (Deelen et al., 2018). Specifically, 70% of Dutch youths aged 12–17, 57% of adults aged 18–64, and 30% of older adults weekly participated in sports (Duijvestijn et al., 2021). However, compared to numerous studies on starting sports, the determinants of stops are understudied. Undeniably, for lifelong sports promotion, understanding why people quit sports is as important as understanding why they start.

Most previous studies on sports discontinuation focus on adolescents and highlight the importance of intrapersonal (i.e., psychological and social-demographic characteristics) and interpersonal (i.e., social interactions) factors (Calvo et al., 2010, Balish et al., 2014, Crane and Temple, 2014, Deelen et al., 2018, Cannella et al., 2022). Specifically, people, especially females and lower-educated adolescents, were reportedly more likely to stop sporting in late adolescence (Lunn, 2010, Prins et al., 2013). Also, loss of interest and enjoyment, poor health, and lack of time can reduce sports participation (Farrell and Shields, 2002, Rottensteiner et al., 2013, Crane and Temple, 2014, Gardner et al., 2017, Jenkin et al., 2017, Jenkin et al., 2018, Jenkin et al., 2021, Pedersen et al., 2021). Another factor is the lack of social support, such as parental objection and low peer acceptance (Rottensteiner et al., 2013, Gardner et al., 2017, Schlesinger et al., 2018, Vojvodić et al., 2020). Despite some evidence, existing studies are insufficient to explain sports discontinuance. Some research gaps exist.

Firstly, studies using long-term longitudinal data are lacking. Previous studies are predominantly cross-sectional, typically ignoring the dynamic nature of sports behavior throughout life. Cross-sectional studies only focus on a specific time. It's unclear whether that time is representative for causality analysis. Indeed, some studies are longitudinal (e.g., Sarrazin et al., 2002, Jakobsson et al., 2012), but they mostly consider short periods. Studies based on long-term longitudinal data are needed to develop lifelong strategies.

Secondly, studies based on a life-course perspective and focusing on multiple life transitions are limited. The life-course perspective is an interdisciplinary approach to analyzing behaviors over several years across serval life stages or affected by several life transitions (Mayer, 2009, Engberg et al., 2012). Although prior studies have recognized that time conflicts are important for the decision to quit sports (Rottensteiner et al., 2013, Eime et al., 2015, Crane and Temple, 2014, Jenkin et al., 2018, Jenkin et al., 2021, Pedersen et al., 2021), few studies have explored the relevant life transitions behind them (Van Houten et al., 2015). According to the life-course theory, life transitions, accompanied by changes in personal and social status or identity as well as changes in living environments, may induce changes in sports behavior (Mortimer and Shanahan, 2003). However, such studies are scarce, many of which are qualitative (Mayer, 2009). For example, via interviews, Haycock and Smith, 2014, Haycock and Smith, 2018 found that life transitions impacted sports dropout through their influence on social inequality, Aarresola et al. (2017) reported that unexpected life events (e.g., illness) affected sports discontinuance, and Gatouillat et al. (2020) identified friends’ decision to stop, injury, and location changes as reasons for teenagers to quit sports.

As for quantitative research on related topics, it is usually less in-depth or only focuses on specific periods (e.g., early adulthood). For instance, Engel and Nagel (2011) speculated that sports discontinuance might be influenced by life transitions such as childbirth and job changes but had no further verification. Similarly, Chen et al. (2022) only discussed whether historical sports participation affected current participation, ignoring the specific roles of life transitions. Additionally, Deelen et al. (2018) focused on youths aged 13–21 and found that players who changed schools had a higher probability of discontinuing tennis. Also, concentrating on early adulthood, Van Houten et al., 2015, Van Houten et al., 2019 showed that respondents participated less frequently in sports when stopping full-time education, beginning to work, entering an intimate relationship, and becoming a parent. Due to the dynamic occurrence of life transitions, studies from a life-course perspective involving multiple life transitions and long-term spans are needed to better understand sports discontinuance.

Thirdly, neighborhood attributes and sports motivations that are important determinants of starting sports have received less attention in explaining sports dropout. It should come as no surprise that a particular driver can start a behavior and also end it when a change occurs. More potential determinants should be explored. Currently, existing research has yielded inconsistent results regarding the impact of neighborhood attributes. Some agreed that inadequate sports facilities or unsafe environments were significantly associated with less activity or dropout (Panter et al., 2008, Crane and Temple, 2014, Halonen et al., 2015, Deelen, 2019, Pedersen et al., 2021). Conversely, others believed that neither perceived safe nor the availability of parks and playgrounds significantly affected sports dropout (Vella et al., 2014, Deelen et al., 2018). Therefore, more research is required to verify these findings.

Given the above gaps, this study contributes to the current literature from three aspects. First, this study has collected longitudinal data to explore sports discontinuance over a quite long period, from birth to the survey date. Second, considering the dynamics of life trajectories, this study examines the effects of multiple life transitions. Third, this study hypothesizes that determinants of sports engagement, including neighborhood attributes and motivations for sporting, may also affect sports dropout for a more comprehensive understanding.

**Fig. 1:**
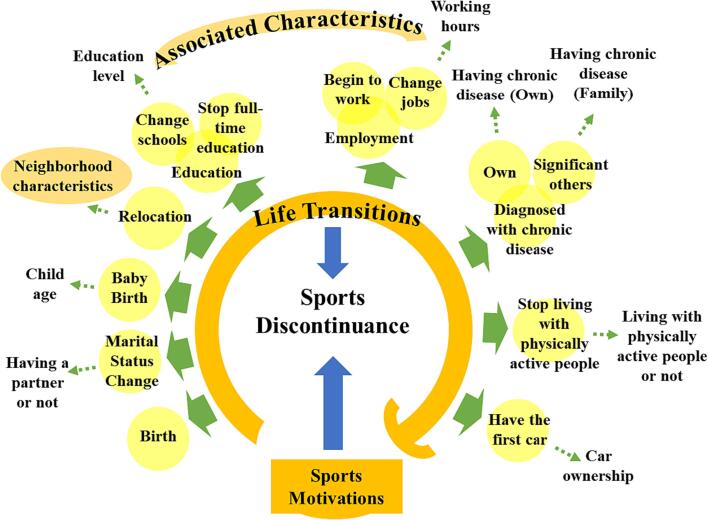
A life-course conceptual framework for this sports discontinuance research (Adult respondents, Netherlands, 2020–2023).

Overall, this study aims to analyze the drivers of discontinuing habitual sports participation from a life-course perspective using retrospective longitudinal data. Specifically, three central questions are focused on: 1) How do major life transitions and associated characteristics influence the decision to quit habitual sports participation? 2) How do neighborhood attributes affect sports discontinuance over the life course? 3) Do the motivations to start sporting also affect the discontinuance over the life course? Habitual sports participation here refers to participation in any form of sports within the scope of the World Sports Encyclopedia (Lipoński, 2003) on a regular basis (at least once a week and lasting more than 6 months). According to the World Sports Encyclopedia (Lipoński, 2003), sports are activities that require physical exertion and skills, have rules and guidelines, and can be competitive. Based on a life-course conceptual framework (Fig. 1), the determinants for examination include socio-demographics, sports motivations, life transitions, and neighborhood characteristics. The results may help develop lifelong interventions to prevent or delay people from quitting sports and promote active living.

## 2 Method

### 2.1 Data collection

Approved by the Ethical Review Board of the TU/e (Reference ERB2019BE2), this study used an online retrospective survey to collect life course data and sports behaviors in the Netherlands from September to October 2020. For decades, retrospective designs have been one of the useful methods for collecting longitudinal data to analyze the relationship between behaviors and life transitions, particularly in the case of long-term life spans (Scott and Alwin, 1998, Moschis, 2019).

With known outcomes, data on historical events, environments, characteristics, and their changes are collected from records or memories (Scott and Alwin, 1998, Ranganathan and Aggarwal, 2018). Despite a risk of memory bias, the retrospective design is comparatively inexpensive, less time-consuming, and can cover a longer time span (Scott and Alwin, 1998, Hirvensalo and Lintunen, 2011). Therefore, the authors developed a retrospective survey to know when respondents changed their participation status in sports and whether these changes co-occurred with life transitions.

### 2.2 Survey design

**Fig. 2:**
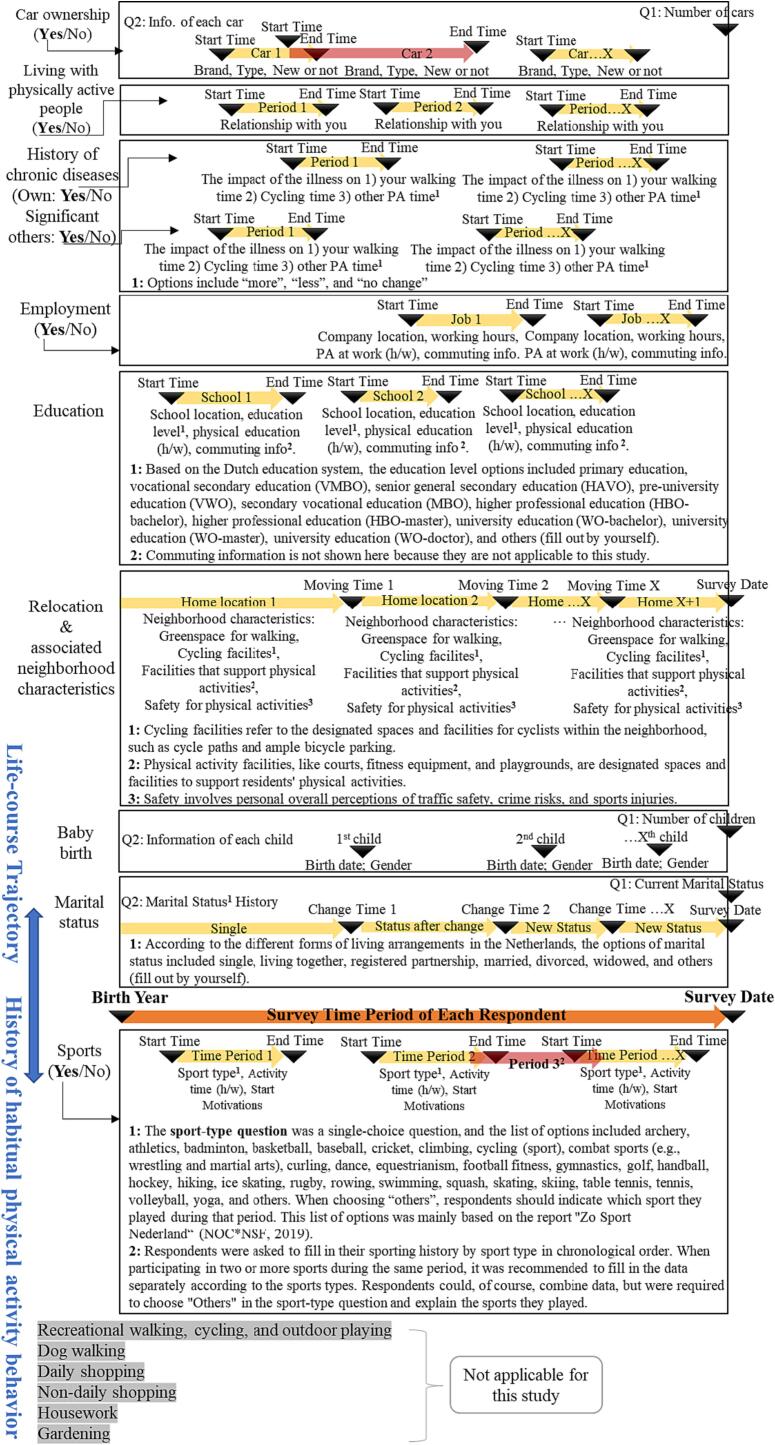
An overview of life trajectory and sports behavior questions in the online retrospective survey (Adult respondents, Netherlands, September-October 2020).

The survey consisted of two sections: life trajectory and history of participation in different forms of physical activity (sports being one of them and the only focus of this study). Fig. 2 contains details of the survey questions.

The questions in the life trajectory section were designed around the major transitions mentioned in the life course theory (Mortimer and Shanahan, 2003). The investigated transitions included 1) birth year, 2) marital status changes, 3) baby birth, 4) relocation and associated neighborhood characteristics, 5) education background, 6) employment history, 7) diagnosis of chronic diseases, 8) living arrangements with physically active people, and 9) car ownership. For each life transition, respondents were asked to indicate whether they experienced this particular event or to answer their current status. According to their responses, they were asked to detail the history of the event and associated characteristics in chronological order from their birth year to the survey date. Additionally, the investigated neighborhood characteristics of each home location included greenspace for walking, cycling facilities, facilities that support physical activities, and safety for physical activities, which were asked via the five-point Likert scale. Respondents were asked to compare each neighborhood they lived in with the average-level Dutch neighborhood and then rate it as few (well below average), below average, average-level, above average, or a lot (well above average).

As for questions on sports, respondents were asked whether they participated regularly (at least once a week) in any form of sports for more than six months. If so, they were asked for further details of each sport they played regularly, including start time, end time, sport type, weekly activity hours, and motivations to start. The options for sport-type questions were mainly based on the report “Zo Sport Nederland” (NOC*NSF, 2019).

**Fig. 3:**
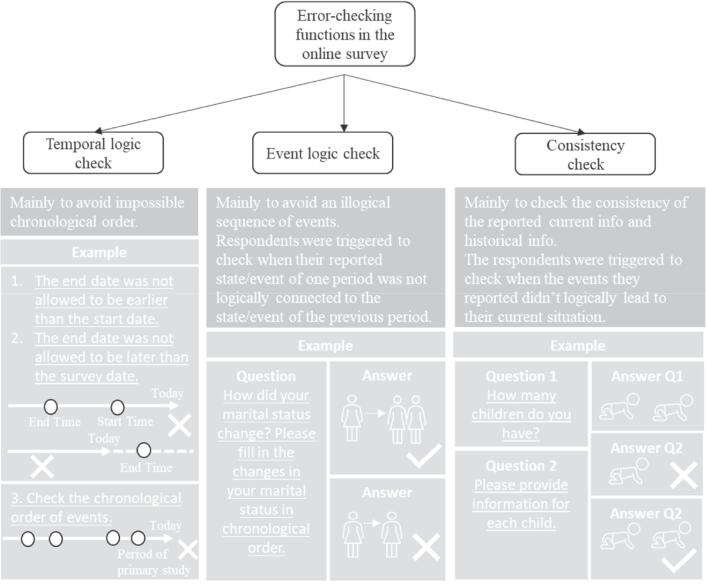
An overview of error-checking functions in the online retrospective survey (Adult respondents, Netherlands, September-October 2020).

The designed survey was created, run, and managed with LimeSurvey, a professional online survey tool. To reduce the possible recall bias and improve data quality, the created online survey system mainly contained three types of error-checking functions to guarantee consistency in responses: 1) temporal logic check, 2) event logic check, and 3) consistency check. Fig. 3 depicts explanations and examples of these functions.

### 2.3 Samples and observations

This investigation collaborated with PanelClix, a national survey company maintaining a representative panel of the Dutch population. PanelClix reaches their panelists in a targeted manner (representative and stratified) based on the characteristics their panelists have provided them. In this study, the qualifying panelists were representative of the Dutch population over the age of 18. Totally, 627 respondents completed the survey, 378 (60.29%) of which participated in sports regularly for more than six months. Only the data from these 378 respondents were used for further analysis.

Sports participation and life transitions are dynamic throughout one's life course and may occur multiple times. To this end, this study integrated the sports participation data with the life-course data to explore whether a respondent stopped sporting under the influence of events changes. Specifically, the process of data integration and observation generation mainly included three steps. Firstly, the sports participation data for each respondent were integrated to identify the periods during which the respondent played sports regularly. In the survey, respondents were asked to review their sports participation by type. Thus, for respondents who had two or more experiences, their answers for the time periods might overlap partially or completely. Since the outcome of this study was whether to stop sporting, it was necessary to integrate temporally overlapping sports participation data for each respondent. Afterward, for each life event, it was checked whether, during some specific period before and after the occurrence of the event, the respondent stopped sporting. Given the possible lag and lead effects, this study assumed that the impact of a baby's birth was within one year before or after, while for other events, six months. Thus, this study included both anticipatory and responsive behavior. Co-occurrence was assumed to indicate that the life event directly or indirectly triggered the dropout. Along with the outcome variable, the status of other explanatory variables was recorded simultaneously. That is, this step generated observations at the timepoints of life transitions. Thirdly, added to these processed data were the data reflecting other occasions of discontinuing sports without the occurrence of a life event. That is, this step generated additional observations at the timepoints of sports discontinuance. In total, 5029 observations were used for further analysis, 10.54% of which stopped active sports participation.

### 2.4 Explanatory variables

**Fig. 4:**
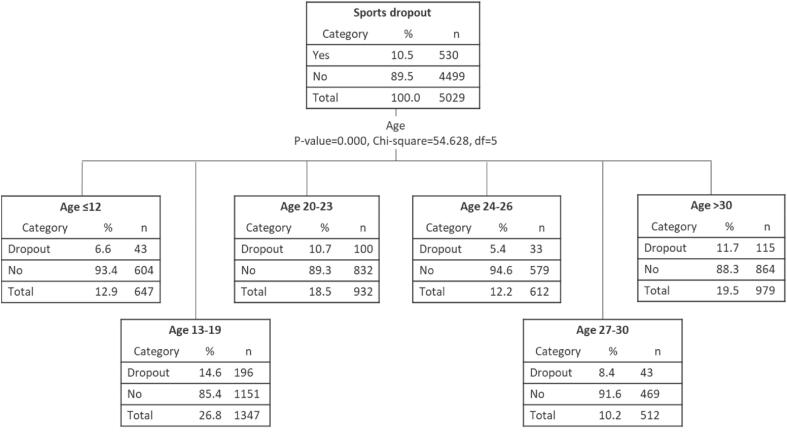
Decision tree results for the explanatory variable age (N = 5029 observations, Netherlands, September-October 2020).

This study considered four types of explanatory variables extracted from the literature on sports participation and dropout: (1) socio-demographics, including age, gender, marital status, education level, weekly working hours, car ownership, child age, having chronic diseases, and living with physically active people, (2) sports motivations, including sports interests, health benefits, and weight control, (3) life transitions, including marital status change, baby birth, residential relocation, changing schools, stopping full-time education, beginning to work, changing jobs, having the first car, and no longer living with physically active people, and (4) neighborhood characteristics, including greenspace for walking, cycling facilities, supportive facilities for physical activities, and safety for physical activities. Table 1 lists all the explanatory variables. Age was divided into six categories based on the result of the decision tree at the observation level (Fig. 4). Neighborhood characteristics were reclassified into two or three levels based on similarity in response distributions. Effect coding was used to represent categorical variables.

**Table 1:** Descriptive statistics of socio-demographic characteristics, sports motivations, life transitions, and neighborhood characteristics (N = 5029 observations, Netherlands, September-October 2020).

Covariates	Levels	PCT (%)	Ratio [Stop sports (%)/Non-stop (%)]
Socio-demographic Characteristics			
Age	≤12	12.87	0.07 [6.65/93.35]
	13–19	26.78	0.17 [14.55/85.45]
	20–23	18.53	0.12 [10.73/89.27]
	24–26	12.17	0.06 [5.39/94.61]
	27–30	10.18	0.09 [8.40/91.60]
	>30	19.47	0.13 [11.75/88.25]
Gender	Female	49.55	0.12 [10.51/89.49]
	Male	50.45	0.12 [10.56/89.44]
Marital status	Have no partner	67.67	0.12 [10.81/89.19]
	Have a partner	32.33	0.11 [9.96/90.04]
Child age	No child	78.78	0.12 [10.60/89.40]
	≤12	17.78	0.11 [9.96/90.04]
	>12	3.44	0.14 [12.14/87.86]
Education level	Primary education or lower	17.26	0.15 [12.79/87.21]
	Secondary education	45.54	0.12 [10.87/89.13]
	Higher education	37.20	0.10 [9.09/90.91]
Working hours	≤20 h/w	58.04	0.14 [12.23/87.77]
	>20 h/w	41.96	0.09 [8.20/91.80]
Car ownership	No	58.18	0.13 [11.59/88.41]
	Yes	41.82	0.10 [9.08/90.92]
Live with physically active people	No	81.29	0.12 [10.76/89.24]
	Yes	18.71	0.11 [9.56/90.44]
Have chronic diseases (Self)	No	90.81	0.12 [10.44/89.56]
	Yes	9.19	0.13 [11.47/88.53]
Have chronic diseases (Family)	No	88.03	0.11 [9.87/90.13]
	Yes	11.97	0.16 [13.79/86.21]

Sports Motivations			
Interest in sports	No	59.22	0.11 [9.84/90.16]
	Yes	40.78	0.13 [11.56/88.44]
Health benefits (Be healthier)	No	57.21	0.13 [11.75/88.25]
	Yes	42.79	0.10 [8.92/91.08]
Weight control	No	88.75	0.12 [10.87/89.13]
	Yes	11.25	0.09 [7.95/92.05]

Life Transitions			
Marital status change	No	92.58	0.12 [11.02/88.98]
	Yes	7.42	0.05 [4.56/95.44]
Baby birth	No	92.92	0.12 [10.83/89.17]
	Yes	7.08	0.07 [6.74/93.26]
Residential relocation	No	84.69	0.13 [11.69/88.31]
	Yes	15.31	0.04 [4.16/95.84]
Change schools	No	75.04	0.14 [12.06/87.94]
	Yes	24.96	0.06 [5.98/94.02]
Stop full-time education	No	91.23	0.12 [11.03/88.97]
	Yes	8.77	0.06 [5.44/94.56]
Begin to work	No	91.47	0.12 [10.87/89.13]
	Yes	8.53	0.08 [6.99/93.01]
Change jobs	No	85.11	0.13 [11.40/88.60]
	Yes	14.89	0.06 [5.61/94.39]
Have the first car	No	93.00	0.12 [10.80/89.20]
	Yes	7.00	0.08 [7.10/92.90]
Stop living with physically active people	No	98.93	0.12 [10.43/89.57]
	Yes	1.07	0.26 [20.37/79.63]

Neighborhood Characteristics			
Greenspace for walking	Few green spaces	5.29	0.08 [7.52/92.48]
	Other levels	94.71	0.12 [10.71/89.29]
Cycling facilities	Few facilities	2.47	0.22 [17.74/82.26]
	Other levels	97.53	0.12 [10.36/89.64]
Physical activity facilities	Few facilities	2.74	0.07 [6.52/93.48]
	Other levels	97.26	0.12 [10.65/89.35]
Safety for physical activities	Below average	6.98	0.16 [13.68/86.32]
	Other levels	93.02	0.11 [10.30/89.70]

### 2.5 Data analysis

This study applied a two-level binary logistic regression model to analyze the determinants of sports discontinuance. This model was chosen for two main reasons. First, the dependent variable of this study was whether a respondent stopped active participation in sports. The responses were binary. Second, this study measured the same respondent at different time points. Observations from the same respondent were less likely to be independent. A two-level binary logistic regression model, which considers the dependency of the data by including a group-level (here, respondent-level) random component in the intercept, is appropriate for this study.

The analysis process involved three steps. Firstly, the multilevel null model with no explanatory variables was built to serve as a reference point for comparison with the final model to see whether the random effects at the group level (here, respondent level) significantly affect the intercept of the dependent variable (here, sports dropout) at the observation level. Secondly, explanatory variables were filtered via univariate analysis. Each explanatory variable was added to the null model for separate analysis to exclude extremely irrelevant variables. Only variables with a p-value less than 0.15 were considered for the final model (Bursac et al., 2008). Finally, all the filtered variables were used as input for the two-level binary logistic regression model. R software was used for estimation (R Core Team, 2022).

Let $y_{\mathrm{ij}}$ denote the binary response for the $i^{\mathrm{th}}$ observation of respondent *j* ($y_{\mathrm{ij}}$ = 1 denotes the occurrence of sports dropout). Let $P_{\mathrm{ij}}=Pr\left(y_{\mathrm{ij}}=1\right)$. Then, the functional form of the two-level binary logistic regression model is:

$$\log\left(\frac{P_{\mathrm{ij}}}{1-P_{\mathrm{ij}}}\right)=\beta_{0}+\beta_{1}^{\prime }x_{ij}+u_{j}$$

where $\beta_{0}$ is the intercept when *x* = 0 and *u* = 0. $x_{ij}$ is a vector of explanatory variables at the observation-level. $\beta_{1}^{\prime }$ is a vector of parameters to be estimated, representing the effects of explanatory variables. $u_{j}$ is the random effects term, capturing the unobserved respondent-level effects. It is assumed to be normally distributed with mean of zero.

In the model-building process, the intraclass correlation coefficient (ICC) is computed to indicate the proportion of the total variance in the outcome variable explained by the grouping structure (Hox, 1995). The equation for ICC is:

$$\mathrm{ICC}=\frac{\sigma_{u}^{2}}{\sigma_{u}^{2}+\left(\pi^{2}/3\right)}$$

## 3 Results

### 3.1 Characteristics of observations

Table 1 indicates descriptive statistics of the observations. To better understand the difference between the group that stopped sports and the group that didn’t stop, a comparison between the two groups was presented. As shown, the percentage of dropouts in the 13–19 group (26.78%) was the highest. The higher the education level, the lower the proportion of discontinuing sports. As for working hours, in 41.96% of observations, people worked more than 20 h/w, and their sports dropout rate was lower than those who worked less. The percentage of having a car was 41.82%, and the ratio of stopping sports to non-stop in these observations was much lower. Besides, people whose children were all over 12 or whose significant others were in poor health only accounted for a small proportion of the observations, but their sports dropout rate was much higher.

Regarding sports motivations, the most popular reason to start sporting was health considerations (42.79%), followed by sports interests (40.78%), and then weight control (11.25%). The proportion of stopping sports among observations that desired to be healthier or control weight was much lower than those without these desires.

Additionally, changing schools was the most observed life event, accounting for about 25% of the total, while stopping living with physically active people was the least, only accounting for 1.07%. For each of the other life events, the observed event changes accounted for 7% to 16% of the total observations. The percentage of discontinuing sports was much higher among the observations that stopped living with physically active people. Conversely, for observations that had a change in other transitions, the rates of discontinuing sports were about 4%-8%, lower than those observed without these transitions.

Concerning neighborhood characteristics, statistical results indicated that the percentage of discontinuing sports in “few cycling facilities” was 17.74%, much higher than that in the other. By contrast, for greenspace and physical activity facilities, this percentage in “few green spaces/facilities” was lower than the other. As for safety, “below average” had the highest ratio of stopping sports to non-stop. For the unmentioned variables, dropout rates within categories were similar, implying small effects.

### 3.2 Results of variables filtering

Table 2 presents the results of univariate analysis, indicating the relationship between each explanatory variable and sports dropout. To exclude extremely irrelevant variables, the cut-off *p*-value was set at 0.15 (Bursac et al., 2008). Estimates showed that the *p*-values of gender, marital status, having chronic diseases (self), living with physically active people, sports interest, cycling facilities, and physical activity facilities were higher than 0.15 and were therefore not considered in the subsequent analysis. Although variables including child age, having chronic diseases (family), living separately from physically active people, greenspace for walking, and safety for physical activities were insignificant at conventional levels, their *p*-values were less than 0.15, meeting the selection criteria. The remaining variables’ *p*-values were less than 0.05, indicating that they were significant for discontinuing sports.

**Table 2:** Results of univariate analysis of all the explanatory variables by two-level binary logistic regression models (N = 5029 observations, Netherlands, September-October 2020).

Covariates		Fixed effects		Random effects:$\sigma_{\mathrm{respondent}}^{2}$
		Coeff.	Sig.	
Socio-demographic Characteristics				
Age	≤12 (Ref.)	−0.408		0.754
	13–19	0.572	0.000 ***	
	20–23	0.156	0.156	
	24–26	−0.652	0.000 ***	
	27–30	−0.066	0.661	
	>30	0.398	0.000 ***	
Gender	Female (Ref.)	−0.022		0.702
	Male	0.022	0.746	
Marital status	No partner (Ref.)	0.012		0.700
	Have a partner	−0.012	0.827	
Child age	No child (Ref.)	−0.117		0.717
	≤12	−0.176	0.140	
	>12	0.293	0.100	
Education level	Primary education or lower (Ref.)	0.334		0.709
	Secondary education	0.007	0.928	
	Higher education	−0.341	0.004**	
Working hours	≤20 h/w (Ref.)	0.198		0.664
	>20 h/w	−0.198	0.000 ***	
Car ownership	No (Ref.)	0.122		0.689
	Yes	−0.122	0.022*	
Live with physically active people	No (Ref.)	0.072		0.702
	Yes	−0.072	0.322	
Have chronic diseases (Self)	No (Ref.)	−0.016		0.700
	Yes	0.016	0.872	
Have chronic diseases (Family)	No (Ref.)	−0.168		0.687
	Yes	0.168	0.059	

Sports Motivations				
Interest in sports	No (Ref.)	−0.088		0.690
	Yes	0.088	0.168	
Health benefits (Be healthier)	No (Ref.)	0.247		0.728
	Yes	−0.247	0.000***	
Weight control	No (Ref.)	0.264		0.718
	Yes	−0.264	0.016*	

Life Transitions				
Marital status change	No (Ref.)	0.497		0.710
	Yes	−0.497	0.000***	
Baby birth	No (Ref.)	0.288		0.711
	Yes	−0.288	0.011*	
Residential relocation	No (Ref.)	0.598		0.718
	Yes	−0.598	0.000***	
Change schools	No (Ref.)	0.412		0.708
	Yes	−0.412	0.000***	
Stop full-time education	No (Ref.)	0.413		0.718
	Yes	−0.413	0.000***	
Begin to work	No (Ref.)	−0.100		0.702
	Yes	0.100	0.015*	
Change jobs	No (Ref.)	0.402		0.706
	Yes	−0.402	0.000***	
Have the first car	No (Ref.)	0.257		0.708
	Yes	−0.257	0.020*	
Stop living with physically active people	No (Ref.)	−0.308		0.692
	Yes	0.308	0.098	

Neighborhood Characteristics				
Greenspace for walking	Few green spaces (Ref.)	−0.243		0.708
	Other levels	0.243	0.076	
Cycling facilities	Few facilities (Ref.)	−0.204		0.686
	Other levels	0.204	0.183	
Physical activity facilities	Few facilities (Ref.)	−0.236		0.696
	Other levels	0.236	0.224	
Safety for physical activities	Below average	0.164	0.092	0.696
	Other levels (Ref.)	−0.164		

Note: 1) Significance codes: p less than 0.001***; p less than 0.01**; p less than 0.05*. 2) The intercept for each model was omitted.

Afterward, the filtered variables were tested for multicollinearity. As shown in Table 3, the variance inflation factors (VIFs) of all the variables were less than 5, indicating that the associated explanatory variable was lowly collinear with the other variables (Vittinghoff et al., 2012). There were no serious multicollinearity problems.

**Table 3:** Multicollinearity test results of the filtered explanatory variables (N = 5029 observations, Netherlands, September-October 2020).

Explanatory variables	VIF
Age: 13–19	2.362
Age: 20–23	1.644
Age: 24–26	1.930
Age: 27–30	2.156
Age: >30	2.862
Child age: ≤12	4.609
Child age: >12	4.149
Education: Secondary education	1.440
Education: Higher education	1.571
Working hours: >20 h/w	1.888
Car ownership: Yes	1.717
Have chronic diseases (Family): Yes	1.037
Health benefits (Be healthier): Yes	1.050
Weight control: Yes	1.051
Marital status change: Yes	1.288
Baby birth: Yes	1.913
Residential relocation: Yes	1.575
Change schools: Yes	2.626
Stop full-time education: Yes	1.511
Begin to work: Yes	1.553
Change jobs: Yes	1.776
Have the first car: Yes	1.526
Stop living with physically active people: Yes	1.050
Greenspace for walking: Below average and above	1.114
Safety for physical activities: Below average	1.102

### 3.3 Results of the two-level binary logistic regression model

A two-level binary logistic regression model was applied to analyze what triggered the discontinuance of sports participation. The log-likelihood value at convergence of the final model was −1228.1, and the corresponding value for the null model without explanatory variables was −1647.6. The likelihood ratio test value was 839.13, much larger than the critical chi-square value with 25 degrees of freedom at any reasonable level of significance. Additionally, the ICC value of the null model indicated that 17.6% of the variance in observation sports discontinuation was attributable to between-respondent differences. Therefore, the consideration of the effects of both observation and respondent level was necessary. Table 4 describes the model results.

**Table 4:** Results of the multivariate analysis of the filtered explanatory variables by the two-level binary logistic regression model (N = 5029 observations, Netherlands, September-October 2020).

Covariates		Coeff.	Sig.
Fixed effects			
Socio-demographic Characteristics			
Age	≤12 (Ref.)	−0.929	
	13–19	0.816	0.000***
	20–23	0.448	0.001**
	24–26	−0.274	0.156
	27–30	−0.008	0.969
	>30	−0.054	0.766
Child age	No child (Ref.)	−0.038	
	≤12	0.117	0.469
	>12	−0.079	0.729
Education level	Primary education or lower (Ref.)	0.701	
	Secondary education	−0.189	0.072
	Higher education (Ref.)	−0.511	0.003**
Working hours	≤20 h/w (Ref.)	0.169	
	>20 h/w	−0.169	0.043*
Car ownership	No (Ref.)	0.165	
	Yes	−0.165	0.054
Have chronic diseases (Family)	No (Ref.)	−0.202	
	Yes	0.202	0.070

Sports Motivations			
Health benefits (Be healthier)	No (Ref.)	0.256	
	Yes	−0.256	0.002**
Weight control	No (Ref.)	0.325	
	Yes	−0.325	0.018*

Life Transitions			
Marital status change	No (Ref.)	1.651	
	Yes	−1.651	0.000***
Baby birth	No (Ref.)	1.542	
	Yes	−1.542	0.000***
Residential relocation	No (Ref.)	1.839	
	Yes	−1.839	0.000***
Change schools	No (Ref.)	1.943	
	Yes	−1.943	0.000***
Stop full-time education	No (Ref.)	1.890	
	Yes	−1.890	0.000***
Begin to work	No (Ref.)	1.691	
	Yes	−1.691	0.000***
Change jobs	No (Ref.)	1.612	
	Yes	−1.612	0.000***
Have the first car	No (Ref.)	1.574	
	Yes	−1.574	0.000***
Stop living with physically active people	No (Ref.)	0.877	
	Yes	−0.877	0.000***

Neighborhood Characteristics			
Greenspace for walking	Few green spaces (Ref.)	−0.339	
	Other levels	0.339	0.041*
Safety for physical activities	Below average	0.422	0.000***
	Other levels (Ref.)	−0.422	
Constant	−14.527	0.000***	

Random effects			
$\sigma_{\mathrm{respondent}}^{2}$: 1.224			
${\mathrm{ICC}}_{\mathrm{final}}$: 0.271			
Marginal $R^{2}$/Conditional $R^{2}$: 0.344/0.522			
AIC/BIC: 2510.1/2686.2			

Note: Significance codes: p < 0.001***; p < 0.01**; p < 0.05*.

The estimated parameters of sociodemographic variables showed that age, education level, and working hours had significant effects on sports discontinuance. The estimated parameters of age were positive. Compared with children under 12, young people aged 13–19 were most likely to discontinue sports, followed by those aged 20–23. The negative parameter of education level indicated that people with a primary education or below were more likely to quit sports than those with a higher education. As for working hours, sports dropouts were more likely to occur when working hours were less than 20 h/w. According to the estimated parameters of sports motivations, individuals with health or weight loss aspirations were less likely to stop regularly sporting.

Moreover, all life events variables showed significant results, albeit the estimated parameters were negative. Nevertheless, the estimated results still illustrated the different effects of various life events on sports dropout. Specifically, the estimated value for stopping living with physically active people was the largest. That is, compared with other life events, the discontinuance of sports participation was most likely to occur when people stopped living with sporty people. Having a baby was in second place. The remaining life events affecting sports discontinuance, in descending order, from largest to smallest, were having the first car, changing jobs, changing marital status, beginning to work, moving home, stopping full-time education, and changing schools.

Furthermore, neighborhood characteristics, including greenspace for walking and safety for physical activities, were significant. The estimated value for greenspace indicated that people were less likely to stop sporting when few green spaces were available in the living environment. Oppositely, the estimation of safety suggested that people were more likely to quit sports when they felt that their neighborhood was unsafe for doing physical activities.

## 4 Discussion and conclusion

This study applied a two-level binary logistic regression model to analyze the determinants of sports discontinuance, including socio-demographics, sports motivations, life transitions, and neighborhood characteristics, from a life course perspective. Data were collected in the Netherlands through an online retrospective survey. Based on a life-course framework, results show that life transitions and associated socio-demographic and environmental characteristics affect sports discontinuance differently. Also, this study implies that starting sports with different motivations may affect the persistence of habitual sports participation. The findings may support sports participants to continue sporting by addressing their barriers over the life course.

An important finding is that people with one or more of the following sociodemographic characteristics are more likely to stop sporting: being 13–23 years old, having a low education level, and working less than 20 h/w. It is consistent with previous research showing a high risk of sports discontinuance in late adolescence, especially among less educated groups (Lunn, 2010, Prins et al., 2013). At the age of 13–19, the most likely period to quit sports, youths may face more activities and heavier academic loads, which can cause difficulties in balancing study, work, life, and sports (Crane and Temple, 2014, Deelen et al., 2018, Persson et al., 2020). This period also coincides with a high incidence of withdrawal from school-organized sports activities, necessitating school-based interventions (Deelen et al., 2018, Espedalen and Seippel, 2022). Additionally, this study suggests that people who start sporting with health or weight loss goals are more likely to stick with it, reflecting the importance of increased awareness of the benefits of sports for sports promotion.

Moreover, this study displays different effects of various life transitions on sports dropout. The end of living with physically active people seems to be the most important event for discontinuation, followed by having a baby. The result illustrates the significant impact of social support. Social factors can be the driving force for sporting. For example, people are more likely to join sports when their significant others do (Farrell and Shields, 2002, Keresztes et al., 2008). Policymakers should act to prevent this force from disappearing. As for having a baby, quitting sports may be due to sports participants taking on more family responsibilities than usual. Active living consultation and services are necessary for families with newborns. Compared with education-related events, work-related events are more likely to cause people to stop sporting. It may hint that it is more difficult for people to prioritize sports on a day-to-day basis when entering a new working environment. Or it may also be related to the location change. As transportation costs increase or sports resources decrease, people are more likely to discontinue sports. Policymakers could consider developing work-based interventions.

Besides, people are more likely to stop sporting in unsafe neighborhoods, but less likely to stop sporting in neighborhoods with insufficient greenspace. Consistent with many previous studies (e.g., Deelen, 2019), safety issues may reduce people’s sports participation as they instinctively avoid dangers. Regarding greenspace, it can be explained that neighborhoods without sufficient greenspace usually provide residents with fewer opportunities for other recreational activities (Sugiyama et al., 2013, Feng et al., 2021). It may partly result in residents regularly playing sports at sports centers and clubs. Combined with other conclusions of this study, neighborhood-based strategies should be developed according to different environmental characteristics and population composition.

Note that this study has some limitations. First, this study collected life-course and sports behavior data retrospectively. Although the possible bias caused by recall has been minimized, the risk is inevitable. Second, respondents were asked to assess neighborhood characteristics by comparison with a typical Dutch neighborhood. It may be a bit general since the Netherlands has many “average-level” neighborhoods. Such self-reported assessments may slightly reduce the sensitivity of the analysis. Additionally, limited by the sample size, this study didn’t further subdivide life transitions when analyzing their relationship with sports discontinuance. Further research is needed to gain insights into subcategories of life transitions.

Despite these limitations, this study expands the evidence base to understand the discontinuance of habitual sports participation. It may assist multidisciplinary practitioners in sports research and practice to create a more supportive social and physical environment for active living. This study suggests that the dynamic nature of sports behavior and the role of life transitions deserve more attention for theoretical development. Also, more potential determinants, such as those to start sports, may have been overlooked. Exploring new determinants remains crucial to enrich existing theories. Since the decision to join or quit sports is complex, dynamic, and even personal, policies and interventions should evolve to be dynamic, lifelong, and tailored.

## CRediT authorship contribution statement

**Xiaoyue Chen:** Conceptualization, Methodology, Investigation, Data curation, Software, Writing – original draft. **Astrid Kemperman:** Conceptualization, Methodology, Investigation, Writing – review & editing, Supervision. **Harry Timmermans:** Conceptualization, Methodology, Investigation, Writing – review & editing, Supervision.

## Declaration of Competing Interest

The authors declare that they have no known competing financial interests or personal relationships that could have appeared to influence the work reported in this paper.

## Data Availability

The data that has been used is confidential.
